# Suspected Radiation-Induced Osteosarcoma in a Domestic Shorthair Cat

**DOI:** 10.1155/2020/1874342

**Published:** 2020-01-03

**Authors:** Natalie Swieton, Stephanie G. Nykamp, Valérie J. Poirier, Shannon Wainberg, Michelle L. Oblak

**Affiliations:** ^1^Atlantic Veterinary College, University of Prince Edward Island, Charlottetown, PE, Canada C1A 4P3; ^2^Ontario Veterinary College, University of Guelph, Guelph, ON, Canada N1G 2W1

## Abstract

A 3-year-old, male neutered domestic shorthair cat, presented for acute onset tail paresis. He was diagnosed with a spindle cell tumour at the level of L7-CD1 and treated with course fractionation radiation therapy. Three years following radiation therapy, the cat developed chondroblastic osteosarcoma of the pelvis, suspected to be secondary to radiation therapy. Hemipelvectomy was performed and the cat was treated with radiation therapy for remaining gross disease. The cat was euthanized 127 days post-operatively due to suspected metastatic disease. Development of radiation-induced tumours should be considered as a rare late complication in cats undergoing radiation therapy.

## 1. Introduction

Primary bone tumours are rare in cats, occurring with an incidence of 4.9 of 100,000 individuals, with osteosarcoma (OSA) comprising 70–80% of these tumours [1, 2]. These tumours can arise from any bone but are slightly more common from appendicular than axial skeletal sites, particularly the proximal humerus, tibia, and distal femur [1]. Affected cats are usually of a mean age of 8–10.7 years, and there is no apparent sex predisposition [2, 3]. Despite their histological similarity, the metastatic rate of OSA in cats is 5–10%, markedly lower than dogs [1, 2]. Development of OSA has been described to occur at the site of healed fractures, device implantation, pre-existing bone disease, and previous irradiation [4–8].

Proposed mechanisms for radiation-induced oncogenesis include genomic instability induced by free radicals and osteodysplasia secondary to osteoblast depletion and impaired vascular supply [8]. Radiation-induced bone tumours have been infrequently reported in humans and dogs [8–10]. In humans, radiation-associated sarcomas comprise up to 5.5% of all sarcomas [11]. In veterinary literature, one retrospective analysis described radiation-induced bone tumours in 8% of dogs treated at appendicular sites with definitive orthovoltage radiation doses totalling 40–51 Gy [12]. No such cases have been reported to date in cats. To the authors' knowledge, this case report describes the first known case of a suspected radiation-induced OSA in a cat.

## 2. Case Presentation

A 3-year-old castrated male domestic shorthair cat was initially referred to the Ontario Veterinary College Health Sciences Centre (OVCHSC) for evaluation of a 1-week history of progressive tail paresis. The patient was a previously stray cat and had tested positive for feline immunodeficiency virus (FIV). Physical examination performed at the time of presentation was unremarkable. Neurological evaluation revealed normal proprioceptive positioning of all four limbs, with absent cutaneous trunci reflexes on the right side, reduced perineal reflex, and markedly decreased tail tone. The lesion was localized between the lumbosacral junction and third sacral spinal cord segment.

Magnetic resonance imaging (MRI) of the lumbosacral spine with contrast (Multihance; Bracco imaging Canada, Montreal, Quebec, Canada) using a 1.5 Tesla device (Signa Explorer; General Electric Healthcare, Waukesha, Wisconsin, USA) identified a homogenously contrast-enhancing mass within the spinal canal, which extended from the caudal third of the seventh lumbar vertebral body to the first caudal vertebral body. The iliosacral lymph nodes were mildly enlarged, more notably on the left. The region of the ischium and pubis was not included in the imaging. Fine needle aspirates of the spinal cord lesion were taken. The samples were of fair quality and had occasional clusters of plump monomorphic spindle cells with wispy grey indistinct cytoplasm. These cells had minimal anisocytosis and anisokaryosis up to two fold. Cytological interpretation was a spindle cell tumour with the two main differential diagnoses being a peripheral nerve sheath tumour and meningioma.

Thoracic radiographs revealed no evidence of metastasis. Computed tomography (CT) scan of the pelvic region was performed for radiation planning with contrast using a 16-slice CT scanner(BrightSpeed/Optima; GE Healthcare, Milwaukee, WI, USA) and identified a healed malunion fracture of the left ischium. The spinal cord mass was not identified on CT. Palliative radiation therapy was elected and the patient was treated with a tapering dose of prednisolone (starting at 1 mg/kg orally (PO) q24h) for 42 days. Course fractionation therapy was administered at five weekly doses of 6 Gy from L6-Cd7. Total dose administered was 30 Gy and the patient was treated with a 5 fields intensity modulated and image guided technic (IMRT/IGRT). Tail function returned to normal throughout the course of treatment and was fully functional by the third week.

Thirty-six months after the end of radiation therapy, the patient returned to the OVCHSC for assessment of a 2-week history of left pelvic limb lameness and 5-day history of obstipation. Physical examination revealed mild discomfort on abdominal palpation and a moderate amount of firm faeces within the colon. A large, firm intrapelvic mass was identified rectally, with narrowing of the pelvic canal. Complete blood count (CBC)(Advia 2120 Hematology Analyzer; Siemens Healthcare Ltd., Oakville, Ontario, Canada) and biochemistry profile (Cobas 6000 C501Biochemistry Analyzer; Roche Diagnostics GmbH, Mannheim, Germany) were unremarkable. Three-view abdominal radiographs identified a moderate amount of faeces within the descending colon. The previously described oblique fracture of the left ischium was noted. The patient was deobstipated and radiographs were repeated. A mass effect was observed in the pelvic canal resulting in ventral displacement of the colon.

**Figure 1:**
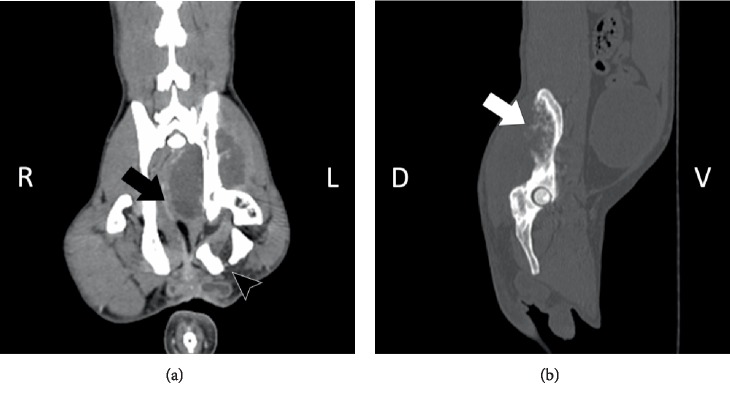
Abdominal computed tomographic images of the cat in bone window dorsal plane post-contrast (a), and sagittal plane (b). A mass can be observed extending into the pelvic cavity with displacement and compression of the rectum to the right (black arrow). Moth-eaten bone less and thin irregular periosteal new bone formation of left ischium is apparent (white arrow). Also note the presence of a chronic left ischium fracture (black arrowhead).

A CT scan of the abdomen was performed under general anesthesia pre- and post- administration of iodinated contrast (Figure 1). The patient was premedicated with hydromorphone (0.05 mg/kg intramuscularly (IM)) and acepromazine (0.03 mg/kg IM). General anesthesia was induced with propofol (5 mg/kg intravenously (IV)). The patient was intubated with a size 5.0 cuffed endotracheal tube and maintained with isoflurane.

**Figure 2:**
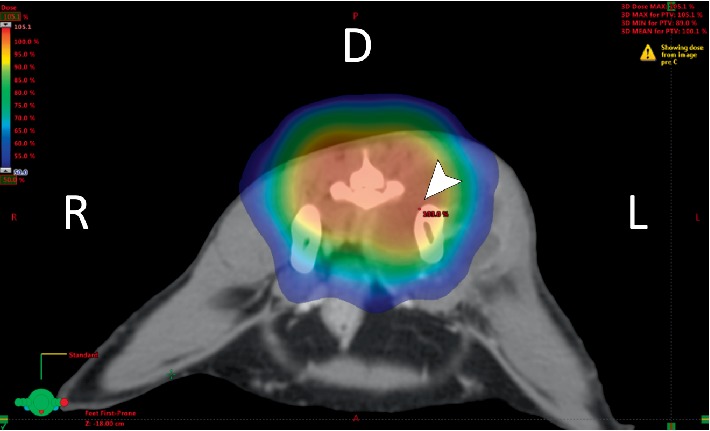
Color wash of radiation dose distributions for treatment of a spindle cell tumour of the spinal cord of the cat using intensity modulated and image guided technic (IMRT/IGRT). Dose color wash is superimposed on a standard window transverse plane post-contrast CT image at the level of the iliac body taken 158 weeks after the last dose of radiation. The high dose is seen in red and low dose in blue. Note the presence of the left ilium and mass within the high dose irradiation field (arrowhead).

CT imaging revealed a large mass with irregular margins and a hypodense nonenhancing central area (Figure 1). The mass extended medially into the pelvic cavity resulting in marked compression and right-sided displacement of the rectum. Moth eaten osteolysis of the left ilium, with thin irregular periosteal new bone formation along the left ilium, cranial left pubis and cranial left ischium was also noted. Bilaterally, the inguinal lymph nodes were mildly enlarged and a mildly enlarged lymph node was present ventral to the sixth lumbar vertebra. The previous radiation plan was evaluated and it was confirmed that the ilium and sacrum were within the field of previous irradiation (Figure 2).

CT imaging of the thorax and pelvis pre and post contrast administration was performed 16 days later for staging and surgical planning. The aggressive mass was larger than previously noted with more obvious involvement of the sacroiliac joint. There was mild enlargement of the left medial iliac lymph node. The remainder of findings were unchanged. CT imaging of the thorax identified a focal area of soft tissue density, measuring approximately 0.7 × 0.6 cm, within the cranial sub-segment of the left cranial lung lobe, which was concerning for metastasic disease.

A left total hemipelvectomy was elected for mass cytoreduction, and to relieve the obstipation caused by the mass. Under the same general anesthetic episode, epidural anesthesia was provided using a combination of hydromorphone (0.02 mg/kg) and bupivacaine(1 mg/kg). A femoral nerve block was also administered with bupivacaine (0.08 mg/kg). Cefazolin (22 mg/kg IV) was given 30 min before first skin incision and every 90 min thereafter.

Sterile gauze was packed into the rectum and a purse string suture pattern was placed within the anus to reduce contamination and increase visualization of the rectum intra-operatively. A urinary catheter (Rusch Pediatric Foley Catheter; Teleflex Medical, Kamunting, Malaysia) was also inserted to make the urethra more readily identifiable. The patient was positioned in right lateral recumbency and a wide area around the left pelvic limb and pelvis was clipped and aseptically prepared.

A semi-circular incision was made on the lateral aspect of the left pelvic limb overlying the middle third of the femur and extended medially. Musculature was transected using a combination of sharp dissection and electrosurgery (ValleyLab ForceTriad Energy Platform; Medtronic, Minneapolis, MN, USA). The cranial sartorius muscle, quadriceps muscles, and remainder of the lateral muscles of the femur were transected proximal to the patella. On the medial aspect of the limb, the pectineus, caudal belly of the sartorius, and gracilis were transected. The femoral artery was double ligated using 3-0 polydioxanone (PDS II; Ethicon, Markham, Ontario, Canada). The medial circumflex femoral vessels were isolated, ligated, and transected. The amputation was continued on the lateral limb by transecting the biceps femoris muscle. The biceps femoris was retracted, and the underlying sciatic nerve was identified and locally infiltrated with bupivacaine prior to transection. The iliopsoas muscle was transected at its insertion on the lesser trochanter. The semimembranosus, semitendinosus, and the adductor muscles were transected at the level of the proximal third of the femur. The adductor magnus was preserved for closure. The gluteal muscles were transected close to their insertion on the greater trochanter. The external rotator muscles and quadratus femoris muscle were severed at their attachments around the trochanteric fossa.

A well-demarcated firm multilobulated mass was found invading into the ilium, crossing the sacroiliac joint, and extending into the surrounding musculature. An oscillating saw was used to cut through the pelvic symphysis. An osteotome and mallet were used to sever the sacroiliac joint and remove the entire hemipelvis. A small amount of gross disease was observed remaining in the sacrum. The surgery site was copiously lavaged with warm sterile saline. Ligating clips (Ligaclip EXTRA; Ethicon Endo-surgery LLC, Guaynabo, Puerto Rico) were placed to denote the perimeter of the surgical site for future radiation treatment.

The adductor and gluteal muscles were used to close the pelvic defect using simple interrupted sutures with 3-0 polydioxanone. The deep subcutaneous tissues were closed using a simple continuous pattern with 3-0 poliglecaprone (Monocryl; Johnson and Johnson, Markham, Ontario, Canada). A wound diffusion catheter (Mila International Inc, Florence, KY, USA) was placed within the subcutaneous tissue, exiting dorsally to the incision, anchored with a finger trap suture using 3-0 polypropylene (Prolene; Johnson and Johnson, Markham, Ontario, Canada) and infused with bupivacaine(0.85 mg/kg). The skin was closed in a cruciate pattern using 3-0 polypropylene. An adherent dressing was applied over sterile gauzes to protect the surgical site. A soft rubber esophagostomy tube (Willy-Rusch, Kemen, Germany) was also placed.

The patient made an uneventful recovery from surgery and was defecating and urinating normally within 2 days post-operatively. He was discharged to the care of his owners at day 4 post-operatively with buprenorphine (0.01 mg/kg oral transmucosally q8–12h) for 5 days. Amoxicillin-clavulanate potassium (10 mg/kg q12h) and meloxicam oral suspension (0.1 mg/kg q24h) were also administered through the esophagostomy tube for 8 days and 5 days respectively.

The amputated limb, hemipelvis, and left popliteal lymph node were submitted for histopathological analysis. The mass on the pelvic section was microscopically multimodular, partially encapsulated, and well demarcated. Neoplastic spindle cells were arranged in streams producing osteoid and islands with dense central chondroid differentiation. Neoplastic cells had small amounts of eosinophilic cytoplasm, 1 to 2 nuclei, and up to 3 prominent nucleoli. There was three-fold anisocytosis and anisokaryosis with a mitotic figure of 27 per 10 400x fields. Approximately 50% of the mass was necrotic and characterized by hypereosinophilic streams of neoplastic cells with loss of cellular detail and lacunae containing hypereosinophilic cells. The left pelvis contained tissue histologically similar to the previously described mass and was comprised of neoplastic spindle cells producing regionally variable amounts of osteoid, chondroid, and fibrous connective tissue. These features were consistent with chondroblastic OSA. Analysis of the left popliteal lymph node revealed a suspected neoplastic cell embolus in a dilated lymphatic vessel indicative of lymphatic spread of the OSA.

Due to the presence of residual disease in the pelvis and suspicion of lymphatic spread, the cat was treated with another course of coarse fractionation radiation therapy for remaining gross disease with 5 daily fractions of 4 Gy, with a total dose of 20 Gy with a 5 fields IMRT/IGRT. A soft opaque nodule superimposed with the cranial lung lobes was noted on thoracic radiographs 82 days post-operatively and was suspicious of progressive metastasis. The patient was euthanized 127 days post-operatively due to declining quality of life from oliguria, obstipation, and reduced pelvic limb function. Abdominal radiographs performed prior to euthanasia revealed ventral compression of the colon by a mass originating from the sacrum.

## 3. Discussion

This case report describes a unique occurrence of an OSA, which developed in the pelvis almost 3 years following radiation therapy for a primary spinal tumour. The young age of the patient, occurrence of two separate neoplasm types, site of development, and aggressive nature of the OSA make this case unusual.

The pelvic OSA described in this case is suspected to be a result of irradiation of the underlying pelvis while targeting the L7-S1 spindle cell mass. The mass fulfills four of five described criteria of a radiation-induced OSA, namely, (i) it developed from an area within the irradiated field [8, 13] (Figure 2), (ii) it developed in bone not known to have a lesion at the time of radiation therapy [8, 13], (iii) it was histologically diagnosed as an OSA which appeared distinct from the initial mass [8, 13], and (iv) the tumour has a low frequency of occurrence at this site [1, 13, 14]. With an asymptomatic latency period of 2.8 years, it does not fulfill the four to five year latency criteria outlined for radiation-induced tumours in humans [8, 13]. It may be reasonable to assume that this latency period may not translate to cats due to their shorter lifespans and immunosuppression due to FIV [8, 15]. Doses of 3.5 to 5 Gy/fraction have been described in reports of radiation-induced OSA in dogs [8, 13]. The radiation dose used in this case was 6 Gy/fraction.

Confirmation of this hypothesis would require histological assessment of the spinal tumour, however risk of iatrogenic neurological damage precluded biopsy and a necropsy was not elected. The most likely differential diagnoses for the spinal spindle cell tumour based on cytology were, peripheral nerve sheath tumour and meningioma. Both of these tumour types have a low propensity for metastasis and only peripheral nerve sheath tumours display locally invasive behaviour in cats [16–18]. Based on the CT images, it is less likely that the pelvic mass was a recurrence or metastasis of the previous spindle cell tumour as the mass was large in size and not adjacent to the spinal cord or centered on L7-S1. Had the pelvic OSA arisen from the spinal cord tumour, we would have expected to see evidence of aggressive behaviour displayed by the spinal mass such as local recurrence or bone destruction in that region [19]. There was no evidence of a spinal cord tumour on the preoperative CT scan for the pelvic mass. However, the possibility that the tumour arose independently, incidentally at a location within the site of radiation cannot be entirely excluded [13].

FIV has been reported to increase likelihood of development of neoplastic disease five to six fold [15]. The most common neoplasm associated with FIV positive cats is B-cell lymphoma, however other lymphoma types, myeloproliferative leukemia, squamous cell carcinoma, fibrosarcoma, mastocytoma, mast cell tumours, and mammary tumours have also been described in infected cats [20, 21]. FIV is speculated to induce oncogenesis through impairment of immune surveillance mechanisms responsible for removal of neoplastic cells, although a few isolated cases show evidence of a more direct mechanism via clonal integration into proto-oncogenes [20]. There is scant evidence supporting preferential development of spindle cell tumours in FIV positive cats; one report of periosteal fibrosarcoma exists [20]. An association between FIV status and OSA development has also not been identified [20]. As such, it is unknown whether the FIV status of our patient was related to the development of these two separate neoplasm types.

Radical surgical excision is the preferred treatment method for feline OSA and curative outcome can often be achieved with clean margins due to the low metastatic rate [1, 2, 22]. Prognosis for appendicular OSA is better than axial OSA, with a mean survival time of 64 months and 6 months, respectively [1]. This difference may be attributed to anatomic limitations posed on surgical excision of axial tumours [1, 2, 14]. Despite the high rate of local recurrence, partial resection of axial OSA has improved survival rates [2]. While surgical excision is the mainstay of treatment, a few case reports implicate radiation and chemotherapy as useful adjuncts [2].

Radiation-induced tumours are a well-recognized entity in humans, and some experimental and clinical reports exist in dogs [12, 13, 23–25]. These tumours generally carry a poor prognosis [26]. Previously published data identified central tumour site location and incomplete surgical remission as negative prognostic factors overrepresented in human radiation-induced sarcomas [26]. The ways this unique subset of secondary tumours behaves differently from sporadically occurring counterparts has not been well characterized [25]. In our case, the presence of suspected metastases at 82 days post-operatively suggested aggressive tumour behaviour. Previous reports of feline OSA had a low metastatic rate of 5-10%, however these values are based on a few studies and may not be representative of axial sites [2]. Higher mitotic index has been associated with a poorer prognosis in feline OSA cases [2]. The mitotic index in this case fit within those values previously reported for primary OSA in this species [2]. Similarly, radiation-induced tumours in dogs and humans microscopically resembled sporadic cases [8]. Additional information is needed to better understand the typical histological and clinical behaviour in primary and secondary feline OSA cases.

Hemipelvectomy is indicated in cases necessitating an aggressive surgical approach, as in malignancies affecting the proximal femur, pelvis, or adjacent soft tissue structures [26]. In tumours that extend beyond the midline of the pelvis, sacrum, or vertebrae, hemipelvectomy is performed with the aim of debulking or palliation [27, 28]. Complete resection in these cases would risk injury to regional gastrointestinal, urogenital, and nervous structures [27, 28]. In subtypes of hemipelvectomy entailing limb amputation, excellent functional outcomes can be achieved similar to pelvic limb amputation alone [29, 30]. A retrospective case series evaluating hemipelvectomy in 84 dogs and 16 cats found that all patients were ambulatory within 24 hours post-operatively [30]. Consistent with previous reports, hemipelvectomy was well tolerated by our patient and was successful in alleviating obstipation. The cat was able to ambulate, urinate, and defecate within 2 days and was discharged 4 days post-operatively. Complications of the procedure, namely substantial intra-operative hemorrhage, wound dehiscence, abdominal herniation, decubitis ulcer formation, incontinence, and neurological deficits in the remaining pelvic limb, were not observed in our patient [29].

This is the first known report of a suspected radiation-induced OSA in a cat. It additionally demonstrates hemipelvectomy as an effective and well-tolerated method for palliative management of tumours when complete resection is not feasible [30]. Based on this case, development of radiation-induced tumours should be considered as a rare late complication in cats undergoing radiation therapy. This information may prove valuable as radiation therapy becomes a more commonly used treatment option for pet owners.
